# Effects of lighting environment on the degeneration of retinal ganglion cells in glutamate/aspartate transporter deficient mice, a mouse model of normal tension glaucoma

**DOI:** 10.1016/j.bbrep.2021.101197

**Published:** 2022-01-05

**Authors:** Tsutomu Ohashi, Kazuhiko Namekata, Xiaoli Guo, Atsuko Kimura, Chikako Harada, Takayuki Harada

**Affiliations:** a Ohashi Eye Center, Sapporo, Japan; b Visual Research Project, Tokyo Metropolitan Institute of Medical Science, Tokyo, Japan

**Keywords:** GCL, ganglion cell layer, GLAST, glutamate/aspartate transporter, IOP, intraocular pressure, IRL, inner retinal layer, mfERG, multifocal electroretinogram, ONL, outer nuclear layer, RGC, retinal ganglion cell, Lighting environment, Retinal ganglion cell, Neurodegeneration, GLAST deficient mice, Glaucoma

## Abstract

Lighting conditions may affect the development of retinal degenerative diseases such as macular degeneration. In this study, to determine whether the lighting environment affects the progression of degeneration of retinal ganglion cells (RGCs), we examined glutamate/aspartate transporter (GLAST) heterozygous (GLAST^+/-^) mice, a mouse model of normal tension glaucoma. GLAST^+/-^ mice were reared under a 12-h light-dark cycle (Light/Dark) or complete darkness (Dark/Dark) condition after birth. The total RGC number in the Dark/Dark group was significantly decreased compared with the Light/Dark group at 3 weeks old, while the number of osteopontin-positive αRGCs were similar in both groups. At 6 and 12 weeks old, the total RGC number were not significantly different in both conditions. In addition, the retinal function examined by multifocal electroretinogram were similar at 12 weeks old. These results suggest that lighting conditions may regulate the progression of RGC degeneration in some types of glaucoma.

## Highlights

• Glutamate/aspartate transporter (GLAST) deficient mouse is a model of glaucoma.
• Dark conditions accelerated the onset of glaucoma in GLAST deficient mice.
• Lighting environment may affect the development of human glaucoma.

## 1 Introduction

Glutamate is an important neurotransmitter for excitatory signals in the retina. In the dark, photoreceptors release glutamate and activate bipolar and horizontal cells, while light suppresses glutamate release [1]. Excessive glutamate in the extracellular space is taken up by glutamate transporters [2]. We previously reported spontaneous degeneration of retinal ganglion cells (RGCs) in mice that lacked the glutamate transporter gene glutamate/aspartate transporter (GLAST; SLC1A3) [3,4]. GLAST is mainly expressed in Müller glial cells, and production of glutathione, a tripeptide of glutamate, cysteine, and glycine, is decreased in GLAST-deficient mice. Since glutathione has a strong anti-oxidative effect, both glutamate neurotoxicity and oxidative stress may be involved in RGC degeneration in GLAST-deficient mice. On the other hand, intraocular pressure (IOP) in GLAST-deficient mice was not increased compared with wild-type mice, thus these mice may be utilized as a model of normal tension glaucoma [[5], [6], [7], [8]].

Previous studies have reported that light exposure may contribute to the onset of retinal degeneration including macular degeneration and retinitis pigmentosa [[9], [10], [11], [12]]. In fact, light-induced photoreceptor degeneration in rodents has been used as a model for human retinitis pigmentosa [13,14]. However, the effect of lighting conditions on RGCs are not well demonstrated. In the present study, we reared GLAST heterozygous (GLAST^+/-^) mice under normal (12 h light: 12 h dark) or complete dark (24 h dark) conditions after birth. We selected GLAST^+/-^ mice because RGC degeneration is milder compared with GLAST^−/−^ mice, which allows us to examine the degeneration process over a relatively long-term [3]. Considering the high release of glutamate in the dark, RGC degeneration may be accelerated or more severe in dark-reared GLAST^+/−^ mice. We examined the time course of RGC degeneration in GLAST^+/-^ mice reared under normal or complete dark conditions.

## 2 Materials and methods

### 2.1 Experimental animals

All experimental procedures in this study were followed in accordance with the ARVO Statement for the Use of Animals in Ophthalmic and Vision Research. Animal experiments were approved by the Institutional Animal Care and Use Committee of the Tokyo Metropolitan Institute of Medical Science (Approval numbers: 14046 and 16082).

GLAST^+/-^ mice, which were obtained by crossing GLAST^−/−^ mice with C57BL/6J mice, were used [4]. Both male and female C57BL/6 or GLAST^+/-^ mice were divided into two groups: the Light/Dark group and Dark/Dark group. For the Light/Dark group, mice were housed under a 12-hr light-dark cycle (light on at 8:00, light off at 20:00). For the Dark/Dark group, newborn mice were moved into a 24-hr dark system with their mother on the day of birth. Mice were kept in the dark system until sacrificed. Both groups were allowed free access to food and drinking water. All efforts were made to minimize animal suffering and the number of animals used.

### 2.2 Immunohistochemistry

Mice were perfused with ice-cold phosphate-buffered saline, followed by 4% paraformaldehyde in 0.1 M phosphate buffer and the eyes were rapidly enucleated. The right eyes were embedded in paraffin wax, cut into 7 μm thick sections, and stained with hematoxylin and eosin. The left eyes were embedded in Tissue-Tek OCT Compound (Sakura Finetechnical, Tokyo, Japan) and frozen. Retinal sections of 10 μm thickness were cut on a cryostat at −20 °C and examined by immunostaining using antibodies against RNA-binding protein with multiple splicing (RBPMS; 1:500; ABN1376; Merck Millipore, Burlington, MA, USA) and osteopontin (1:1000; AF808; R&D, Minneapolis, MN, USA).

Stained sections were examined using a microscope (BX51; Olympus, Tokyo, Japan) equipped with Plan Fluor objectives (Olympus) connected to a DP73 camera (Olympus). Images were processed and viewed using a DP manager software (v2.2.1.195; Olympus). For quantification, the number of total cells, RBPMS-positive or osteopontin-positive cells in the ganglion cell layer (GCL) was counted from one ora serrata through the optic nerve to the other ora serrata [15] in three sections per mouse. The number of mice examined is shown in each figure legend.

### 2.3 Multifocal electroretinogram (mfERG)

The mfERGs were recorded using a VERIS 6.0 system (Electro-Diagnostic Imaging, Redwood City, CA, USA). Before examination, mice were anesthetized with an intraperitoneal injection of sodium pentobarbital (87.5 mg/kg) and the pupils were dilated with a mixed solution of 0.5% phenylephrine and 0.5% tropicamide. The visual stimulus consisted of seven hexagonal areas scaled with eccentricity. The stimulus array was displayed on a high-resolution black and white monitor driven at a frame rate of 100 Hz. The second-order kernel was analyzed as previously reported [3,4,16].

### 2.4 Statistical analysis

Statistics were performed using a JMP 13 software (SAS Institute, Cary, NC, USA). Data are represented as the mean ± standard error. Data significance was determined using two-tailed Student's *t*-tests, or one-way ANOVA with Dunnett's post hoc test. A p value of less than 0.05 was considered statistically significant.

## 3 Results

### 3.1 RGC degeneration and visual function in GLAST^+/-^ mice reared in the Light/Dark and Dark/Dark conditions

**Fig. 1:**
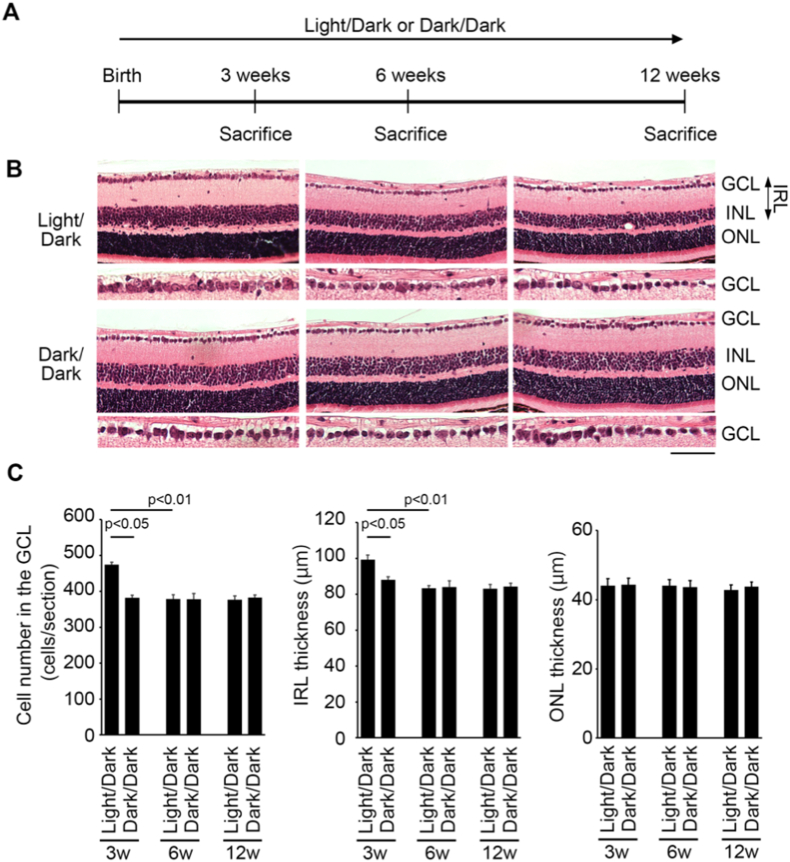
Retinal degeneration in Light/Dark- and Dark/Dark-reared GLAST^+/-^ mice. **A:** Experimental protocols. Mice were reared in a Light/Dark condition or kept in a Dark/Dark environment from birth and sacrificed at 3, 6, and 12 weeks. **B:** Hematoxylin and eosin staining of retinal sections. Scale bar: 100 and 50 μm in the upper and immediately lower panels, respectively. GCL: ganglion cell layer; INL: inner nuclear layer; ONL: outer nuclear layer; IRL: inner retinal layer. **C:** Quantitative analyses of the cell number in the GCL, IRL thickness and ONL thickness. The data are presented as mean ± standard error. n = 3–4 mice per group.

We first examined the progression of RGC degeneration in GLAST^+/-^ mice reared in the Light/Dark and Dark/Dark conditions (Fig. 1A). The retinal structure in the Dark/Dark group was similar compared with that in the Light/Dark group (Fig. 1B). We previously reported that RGC degeneration in GLAST^+/-^ mice starts at 3 weeks old and the RGC number is stable after 5 weeks old [3,4]. Consistent with our previous reports, the cell number in the GCL and the thickness of the inner retinal layer (IRL) were decreased between 3 and 6 weeks old in the Light/Dark group (Fig. 1B and C). The cell number in the GCL and the IRL thickness in the Dark/Dark group were similar to the Light/Dark group at 6 and 12 weeks old. However, at 3 weeks old, the cell number in the GCL and the IRL thickness in the Dark/Dark group were significantly decreased compared with the Light/Dark group (Fig. 1B and C). On the other hand, the thickness of the outer nuclear layer (ONL) was similar in both groups at 3, 6 and 12 weeks old (Fig. 1B and C).

**Fig. 2:**
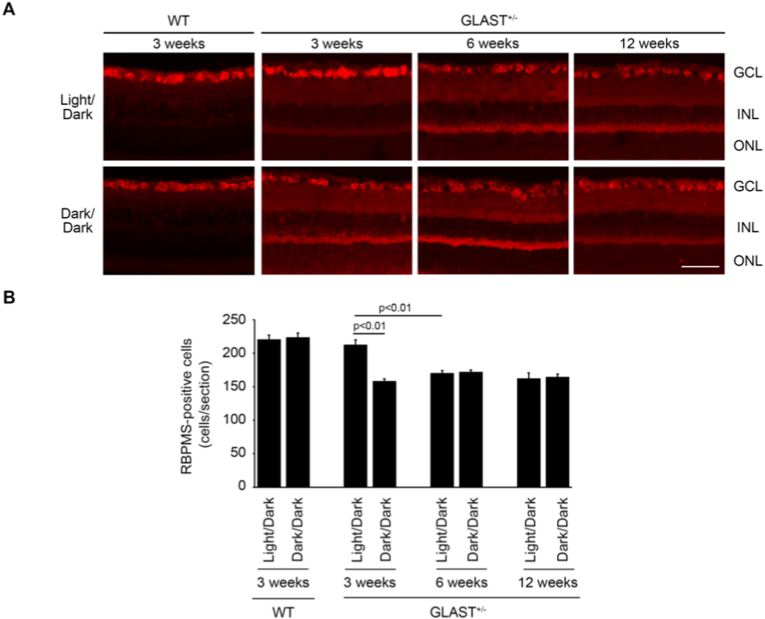
RBPMS-positive cells in Light/Dark- and Dark/Dark-reared wild-type and GLAST^+/-^ mice. **A:** RBPMS immunostaining of retinal sections from WT mice at 3 weeks old, and GLAST^+/-^ mice at 3, 6, and 12 weeks old reared under the Light/Dark or Dark/Dark environment. Scale bar: 100 μm. GCL: ganglion cell layer; INL: inner nuclear layer; ONL: outer nuclear layer. **B:** Quantitative analyses of RBPMS-positive cell numbers in the GCL. The data are presented as mean ± standard error. n = 3–5 mice for Light/Dark group and n = 3–4 mice for Dark/Dark group.

We also carried out immunohistochemical analysis using an antibody against RBPMS, a RGC marker. The number of RBPMS-positive cells in the wild-type mice was similar in the Light/Dark and Dark/Dark conditions at 3 weeks old (Fig. 2). In GLAST^+/-^ mice, the number of RBPMS-positive cells in the Dark/Dark group was significantly decreased compared with the Light/Dark group at 3 weeks, but not at 6 and 12 weeks old (Fig. 2). These results suggest that the retinal structure and the total RGC number in both groups are similar in adult GLAST^+/-^ mice, but not in young GLAST^+/-^ mice.

**Fig. 3:**
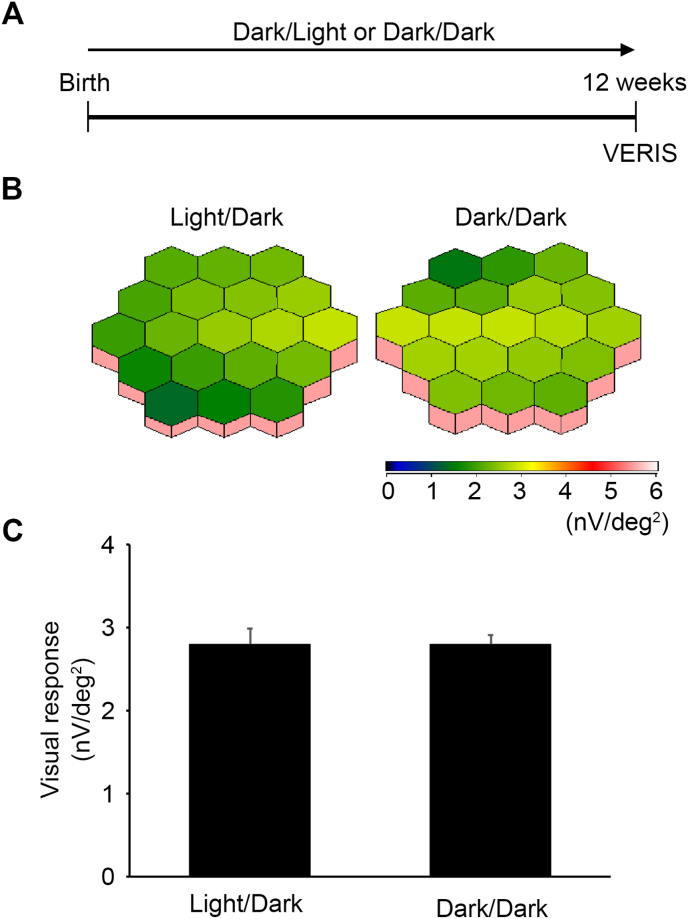
Visual function in Light/Dark- and Dark/Dark-reared GLAST^+/-^ mice. **A:** Experimental protocols. Mice were reared in a Light/Dark condition or kept in a Dark/Dark environment from birth and visual function were measured at 12 weeks old. **B:** Averaged retinal responses demonstrated using three-dimensional plots. **C:** Quantitative analyses of the retinal response amplitude. The data are presented as mean ± standard error. n = 6 mice for Light/Dark group and n = 7 mice for Dark/Dark group.

To confirm this point, we next examined the visual function of GLAST^+/-^ mice by using mfERG. Unfortunately, we could not examine mfERG at 3 weeks due to small eyes, but it was possible at 12 weeks old (Fig. 3A). Consistent with the histological findings, visual responses were similar in both groups (Fig. 3B and C).

### 3.2 Osteopontin-positive αRGCs survive in GLAST^+/-^ mice reared in the Light/Dark and Dark/Dark conditions

**Fig. 4:**
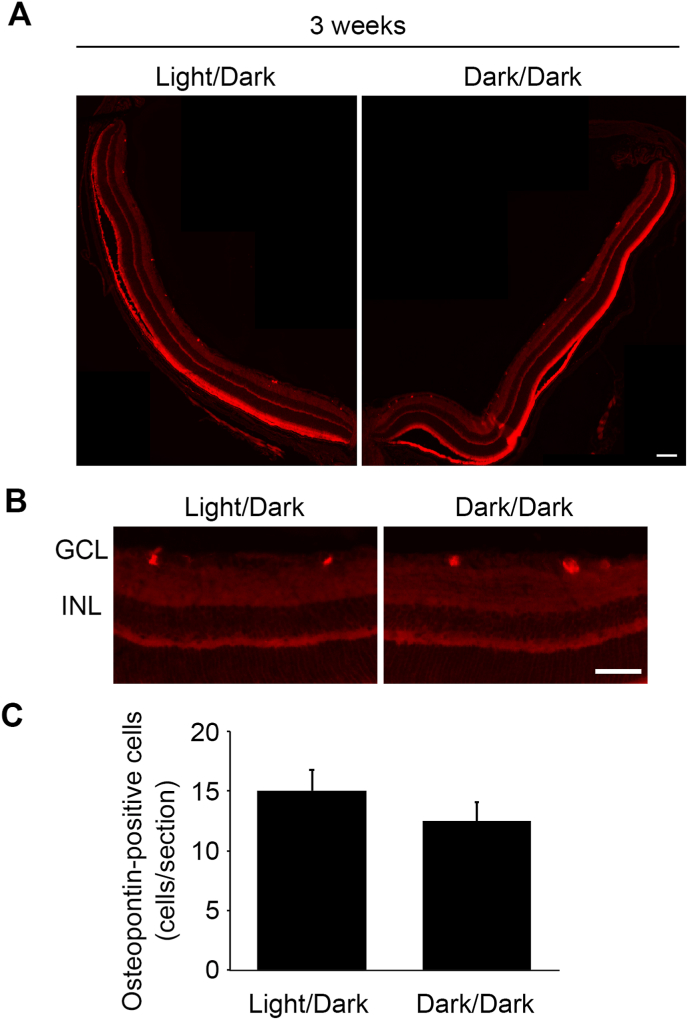
Osteopontin-positive cells in Light/Dark- and Dark/Dark-reared GLAST^+/-^ mice. **A:** Low magnification of retinal sections immunostained with an anti-osteopontin antibody in Light/Dark and Dark/Dark GLAST^+/-^ mice at 3 weeks old. Scale bar: 100 μm. **B:** High magnification of retinal sections immunostained with an anti-osteopontin antibody. Scale bar: 100 μm. GCL: ganglion cell layer; INL: inner nuclear layer. **C:** Quantitative analyses of osteopontin-positive cell numbers in the GCL. The data are presented as mean ± standard error. n = 5 mice for Light/Dark group and n = 4 mice for Dark/Dark group.

We previously reported that osteopontin-positive αRGCs, which are found in less than 10% of the total RGCs, are tolerant to glutamate neurotoxicity and survive in GLAST^−/−^ mice [17]. Since the total RGC number was decreased in GLAST^+/-^ mice in the Dark/Dark group at 3 weeks old (Fig. 1, Fig. 2), we examined whether αRGCs in GLAST^+/-^ mice survive in the Dark/Dark group as well as in the Light/Dark group at 3 weeks old. We carried out immunohistochemical analysis and detected osteopontin-positive αRGCs in the GCL in both groups (Fig. 4A and B). Quantitative analysis revealed that the number of osteopontin-positive cells was similar in both groups (Fig. 4C). These results suggest that RGC subtypes other than αRGCs degenerate faster in GLAST^+/-^ mice in the Dark/Dark group by 3 weeks old.

## 4 Discussion

In the present study, we used GLAST^+/-^ mice as an animal model of RGC degeneration. This model shows glaucomatous phenotypes such as RGC loss, optic nerve atrophy, and visual impairment while maintaining normal IOP [3,4]. Although the early onset of RGC degeneration in GLAST^+/-^ mice is atypical compared with human glaucoma, it may be an advantage to carry out basic experiments in a short span of time [[3], [4], [5], [6], [7], [8]]. We first examined the effects of lighting environment in GLAST^+/-^ mice and found that RGC degeneration started before 3 weeks old in the Dark/Dark condition. One possibility is that glutamate concentration in the retina was chronically upregulated under the Dark/Dark condition compared with the Light/Dark condition. In C57BL6J mice, the Dark/Dark condition has no effect on retinal development and structure, however, this condition may accelerate RGC degeneration in GLAST^+/-^ mice. For further experiments, we may rear GLAST^+/-^ mice in the Light/Dark condition until 3 weeks old, and then divide them into the Light/Dark and Dark/Dark conditions to examine the effects of the Dark/Dark condition on RGC degeneration in more detail.

We previously examined the glutamate concentration in the vitreous, but it was not increased in GLAST^+/-^ mice [3]. We tried to detect glutamate concentration using magnetic resonance spectroscopy i*n vivo* [18,19], but failed because mouse eyes were too small for the methods. We would like to determine the possibility of increased glutamate levels in the mouse eye in the future experiments. On the other hand, as an alternative approach to show that glutamate availability is involved in RGC loss, we are planning to test the effects of glutamate receptor antagonists on RGC loss in GLAST^+/-^ mice.

One may expect more severe RGC degeneration at 6 and 12 weeks old GLAST^+/-^ mice reared in the Dark/Dark condition compared with the Light/Dark condition, but the extent of the degeneration was similar. In addition, the number of αRGCs was similar between the two groups. In mammals, there are more than 40 different subtypes of RGCs that differ in soma size, morphology, dendrite arborization, and electrophysiological functions [[20], [21], [22], [23], [24]]. We previously reported that αRGCs and intrinsically photosensitive RGCs (ipRGCs) are highly tolerant to glutamate neurotoxicity and survive in GLAST^−/−^ mice [17]. These results suggest that the Dark/Dark condition stimulated early RGC degeneration in GLAST^+/-^ mice, but some RGC subtypes including αRGCs were not affected by darkness after 3 weeks old.

In retinitis pigmentosa and macular degeneration, phototoxic damage may stimulate the process of the disease [[9], [10], [11], [12]]. In addition, visual light may have different effects on mitochondrial function and cell survival in RGCs according to wave length [25]. On the other hand, phototoxicities by continuous light exposure were not induced in rat RGCs after transduction of the optogenetic gene mVChR1 using an adeno-associated virus vector [26]. Our results suggest a possibility that darkness may also affect the time course of RGC degeneration in the case that involves glutamate neurotoxicity in the pathogenesis [8]. For example, some missense mutations in the EAAT1 gene (the human homolog of GLAST) may decrease glutamate uptake into Müller cells and increase the rate of RGC death in glaucoma patients [7,27]. Recent studies have reported that glutamate concentration can be examined in human eyes and visual pathways [18,28,29]. Further studies are required to determine whether lighting environment affects the progression of retinal degeneration including glaucoma.

## Declaration of competing interest

The authors declare that they have no known competing financial interests or personal relationships that could have appeared to influence the work reported in this paper.
